# The Role of Fusion in Ant Chromosome Evolution: Insights from Cytogenetic Analysis Using a Molecular Phylogenetic Approach in the Genus *Mycetophylax*

**DOI:** 10.1371/journal.pone.0087473

**Published:** 2014-01-28

**Authors:** Danon Clemes Cardoso, Silvia das Graças Pompolo, Maykon Passos Cristiano, Mara Garcia Tavares

**Affiliations:** Programa de Pós–graduação em Genética e Melhoramento, Departamento de Biologia Geral, Universidade Federal de Viçosa, UFV, Viçosa, Mina Gerais, Brazil; Natural Resources Canada, Canada

## Abstract

Among insect taxa, ants exhibit one of the most variable chromosome numbers ranging from *n = 1* to *n = 60*. This high karyotype diversity is suggested to be correlated to ants diversification. The karyotype evolution of ants is usually understood in terms of Robertsonian rearrangements towards an increase in chromosome numbers. The ant genus *Mycetophylax* is a small monogynous basal Attini ant (Formicidae: Myrmicinae), endemic to sand dunes along the Brazilian coastlines. A recent taxonomic revision validates three species, *Mycetophylax morschi*, *M. conformis* and *M. simplex*. In this paper, we cytogenetically characterized all species that belongs to the genus and analyzed the karyotypic evolution of *Mycetophylax* in the context of a molecular phylogeny and ancestral character state reconstruction. *M. morschi* showed a polymorphic number of chromosomes, with colonies showing 2n = 26 and 2n = 30 chromosomes. *M. conformis* presented a diploid chromosome number of 30 chromosomes, while *M. simplex* showed 36 chromosomes. The probabilistic models suggest that the ancestral haploid chromosome number of *Mycetophylax* was 17 (Likelihood framework) or 18 (Bayesian framework). The analysis also suggested that fusions were responsible for the evolutionary reduction in chromosome numbers of *M. conformis* and *M. morschi* karyotypes whereas fission may determines the *M. simplex* karyotype. These results obtained show the importance of fusions in chromosome changes towards a chromosome number reduction in Formicidae and how a phylogenetic background can be used to reconstruct hypotheses about chromosomes evolution.

## Introduction

Chromosomes are the units of inheritance contained in the nuclei of eukaryotic cells. They can have different sizes, shapes and DNA compositions and there is ample evidence that chromosome changes may promote speciation [1]. A large number of ant species have been studied cytogenetically and they exhibit an enormous diversity of chromosome number, varying from *n = 1* to *n = 60* (reviewed in [2]). This marked variation aroused attention over 35 years ago [3], such that several evolutionary mechanisms and the manner in which this diversity has evolved have been proposed. Among the evolutionary mechanism proposed, rearrangements involving Robertsonian fissions stands out (see [4]–[5]) in the so–called “minimum interaction theory”. According to this theory, the chromosome evolution in ants generally tends towards an increase in chromosome number in order to reduce the risk of deleterious rearrangements [5]. Nevertheless, it has been thought that the typical trend in chromosome increase probably respects certain limits [2].

The growing number of cytogenetic studies in ants have highlighted that chromosome evolution has accompanied genus and species diversification [2]. Thus, karyotype descriptions and their comparative analyses is an important independent tool for taxonomy and understanding chromosome evolution, particularly when relying on phylogenetic tree construction [6]. The ready availability of DNA sequences and advances in molecular phylogenetic analysis has allowed researchers to infer the relationships of ants that can be used in an integrative cytogenetic approach. In addition, sequences of protein–coding nuclear genes have been shown to be useful for resolving phylogenetic relationships within genera and between related species in ants [7].

The Attini tribe belongs to the Myrmicinae subfamily and comprises ants that are known to engage in a symbiosis with a *Basidiomycota* fungus, which serves as their main food source. They are restricted to the New World and are primarily distributed in the Neotropics, where they achieve their greatest diversity. Currently, the tribe comprises more than 230 described species grouped into 14 genera [8]–[9]. Although some systematic studies on Attini tribe have been conducted, information is scarce regarding the majority of the groups and the taxonomy of many species still requires revision. Indeed, recent revisionary studies have permitted the identification of sibling species [10], description of new species [11], and even the description of a new genus [9].

The genus *Mycetophylax* is a small monogynous basal Attini that has recently gained more attention [9], [13]–[15]. About 20 species, subspecies and varieties were coded to the genus *Mycetophylax* [9], although Kempf [16] lists 15 species living in Brazil. Following the taxonomic revision based on morphological systematics, the majority of those species were synonymized and some others were included into *Mycetophylax*, originally belonging to the Attini genus *Cyphomyrmex*. Currently the genus *Mycetophylax* is composed of three valid species, *M. conformis* (Mayr, 1884), *M. morschi* (Emery, 1888) and *M. simplex* (Emery, 1888). However, some issues concerning the occurrence of sibling species within *Mycetophylax* and the status of *M. morschi* belonging to the genus still remain under discussion (Mayhé–Nunes pers. Com.). Analysis of wingless and long–wave rhodopsin led to the recent molecular phylogenetic hypothesis of the *Mycetophylax* genus [17], which was in agreement with the morphological features. Recently, the nuclear content of the three species were estimated by flow cytometry, data that provided noteworthy information at a higher level than the species level [14], but information concerning the *Mycetophylax* karyotype is no longer available.

Thus, the aim of this study was to provide the first characterization of *Mycetophylax* species karyotype, including chromosome number, morphology, heterochromatin location and chromatin AT/GC richness. We discuss the evolutionary dynamics of the karyotypes within the genus in the light of the recently published phylogeny. Additionally, lineage–specific rearrangements leading to different chromosome numbers in *Mycetophylax* were tested using ancestral state reconstruction. For this we used two different, recently developed approaches by Mayrose et al. [18] based on Maximum Likelihood and Bayesian methods in order to propose insights concerning chromosome evolution in ants.

## Materials and Methods

### Biological material and chromosome preparation

**Figure 1:**
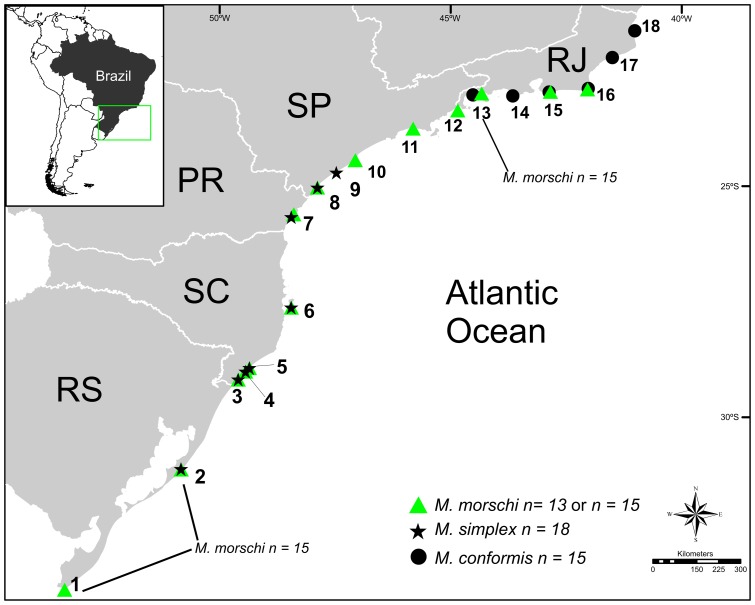
Cytogenetically characterized populations of *Mycetophylax* species across the distribution of the genus along Atlantic coast. Sampling sites and karyotype results. Populations of *M. morschi* with haploid number of 15 chromosomes are indicated. Numbers represent sampling localities as given in the Table S1.

Colonies of the three species were collected from sand dunes throughout their occurrence area along the Brazilian Atlantic coast, from Rio Grande do Sul State to Rio de Janeiro State between December 2009 and March 2011. The colonies of *M. simplex* (19 colonies) were collected on beaches in the States of Rio Grande do Sul, Santa Catarina, Paraná and Rio de Janeiro. *M. conformis* (21 colonies) were collected on beaches in the States of Rio de Janeiro and São Paulo, while *M. morschi* (38 colonies) were collected in all the states mentioned (Figure 1; Table S1 for sampling details). Following collection, the colonies were transported to the laboratory and reared according to the protocol described by Cardoso et al. [13] until brooding occurred. When available, at least ten individuals from each colony were used in cytogenetic analyses. All the ants collected were preserved in ethanol and confirmation of species identification was performed by Rodrigo Feitosa, at the Museum of Zoology of the University of São Paulo (*Museu de Zoologia da Universidade de São Paulo*, MUZSP), where vouchers were also deposited. All species' collections were authorized by the Brazilian Institute for Biodiversity Conservation (ICMBio) by means of a special permit (number 24869–2) recorded by SISBio. Collecting permit was issued to Danon Clemes Cardoso in Brazil.

Metaphase spreads were prepared from the cerebral ganglia of post–defecant larvae, according to protocol proposed by Imai et al. [19]. The cerebral ganglion was dissected in colchicine–hypotonic solution (0.005%) under a stereoscopic microscope, transposed to a new drop of same solution and incubated under light protection for one hour until slide preparation (see reference [19] for detailed procedure). The slide with metaphases were examined under a phase contrast microscope and stained with 4% Giemsa solution in Sorensen's buffer, pH 6.8, to determine chromosome number and morphology. We classified the chromosomes following a modified nomenclature based on the proposed by Levan et al. [20], which is based on four types of centromeric position: acrocentric (A), subtelocentric (ST), submetacentric (SM) and metacentric (M).

### C–banding and Fluorochrome staining

In order to determine the distribution pattern of heterochromatin, the BSG (barium hydroxide/saline/Giemsa) banding technique was performed, essentially following the method described by Sumner [21], with modifications in the duration of treatment with Ba(OH)_2_, as proposed by Pompolo and Takahashi [22]. Sequential fluorochrome staining with chromomycin A3/distamycin A/4′–6′–diamindino–2–phenylindole (CMA3/DA/DAPI) was conducted according to Schweizer [23] in order to characterize CG and AT richness region on chromosomes. The slides were analyzed under an epifluorescence microscope (Olympus BX 60) equipped with a digital camera system (Q color 3 Olympus®). The fluorescent signals were analyzed with different filters: WB filter (450 to 480 nm) for the fluorochrome CMA3 and WU filter (330 to 385 nm) for the fluorochrome DAPI. At least nine and six slides of each species with well-spread metaphases were submitted to C-banding technique and fluorochrome staining, respectively.

### Chromosome evolution analysis

In order to infer and support the patterns and processes underpinning chromosomal evolution in *Mycetophylax* an integrative cytogenetic and molecular phylogeny study was conducted. To determine the direction of chromosomal changes (i.e. fusion versus fission) that occurred in the genus *Mycetophylax*, Attini species with known karyotypes were used as an out–group. Thus, two different methods were performed with the purpose of outlining a chromosome evolution hypothesis for this genus. The software ChromEvol 1.3 [18] was used to infer the chromosome evolution model and haploid ancestral states (chromosome numbers) by Maximum Likelihood and Bayesian methods, relying on a previously phylogenetic hypotheses published by our group [17].

The molecular phylogenetic tree for *Mycetophylax*, on which the haploid ancestral states were inferred in this work, was based on the wingless and long–wave rhodopsin matrix of Cardoso et al. [17]. Sequences of *wingless* and *long–wave rhodopsin* genes were downloaded from GenBank (Table S2) and aligned using Mega 5.0 [24]. Therefore, we reconstructed the Bayesian tree from that study using selected taxa that were cytogenetically characterized or that have their karyotype known, with the same setting parameters and substitution model HKY+G for long–wave rhodopsin and GTR+G for wingless to run MrBayes 3.2.2 [25]. ChromEvol 1.3 was carried out and relied on this reconstructed tree. The program allows the evaluation of eight models of chromosome evolution taking into account: gains and losses of single chromosomes; duplications, whole–genome duplication; and demi–duplication, a mechanism that facilitates the transition from an *n* chromosome to 1.5 *n*. The last feature was not evaluated in our analysis, since it is only widespread and common in plants. Therefore, four models of chromosome evolution that did not consider demi–duplication were carried out. All the parameters were adjusted to the data following the recommendation of Mayrose et al. [18]. The models and their null hypotheses were analyzed with 10,000 simulations and the one that best fit the data set was selected under the Akaike information criterion (AIC).

Subsequently, to define a more complete chromosome evolution hypothesis, the node ages of *Mycetophylax* were estimated. This analysis was performed to determine when the possible splits between lineages occurred, to assess the information of chromosome changes in a geological and evolutionary context. Thus, molecular dating of *Mycetophylax* lineages were estimated using previously reported nuclear clock calibrations for Attini ants [8]. In a matrix for the genes *wingless* and *long*–*wave rhodopsin* downloaded from the NCBI GenBank (see Table S2), we included sequences of cytogenetically characterized *Mycetophylax* species. The nuclear genes matrix was analyzed under a Bayesian framework and uncorrelated lognormal–relaxed clock model in BEAST v. 1.6.1. [26] as described by Rabeling et al. [27].

## Results

### Karyotype analysis and chromosome banding

**Figure 2:**
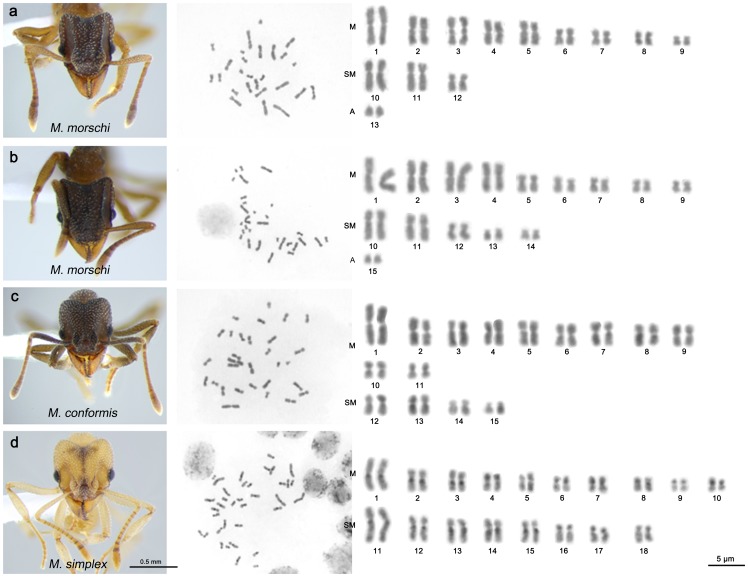
Conventional staining of mitotic cells of *Mycetophylax*. Species images, metaphases and diploid karyotypes of *Mycetophylax morschi* 2n = 26 (a) and 2n = 30 (b), *Mycetophylax conformis* (c) and *Mycetophylax simplex* (d). M =  metacentric, SM =  submetacentric and A =  acrocentric.

The three species of the genus *Mycetophylax* investigated present different chromosome numbers and karyotype morphologies (Figure 2). *M. morschi* showed populations with two distinct chromosome sets. Populations from some beaches of Rio Grande do Sul and Rio de Janeiro State presented diploid chromosome numbers equal to 2n = 30 (*n = 15*) or 2n = 26 (*n = 13*), whereas all the populations from Santa Catarina, Paraná and São Paulo State showed 2n = 26 (Figure 1, 2). No hybrids were found. *M. morschi* presented an almost bimodal karyotype that consisted of seven large and six small chromosome pairs for populations with 2n = 26 chromosomes, and six large and nine small pairs in populations with 2n = 30 chromosomes (Figure 2a and b). Both *M. morschi* karyotypes displayed nine metacentric pairs and one acrocentric pair; however *M. morschi* 2n = 26 showed only three submetacentric pairs, whereas specimens with 2n = 30 showed five. In karyotypes with 2n = 26, one strongly and two weakly stained regions were observed in the terminal portion of the long arm of chromosome 3 and chromosomes 2 and 5, respectively. These regions may correspond to secondary constrictions. Similarly, a secondary constriction on chromosome 3 was observed in the karyotype 2n = 30.

In all of the metaphases analyzed, *M. conformis* presented 2n = 30 (*n = 15*) and a chromosome complement composed of eleven metacentric and four submetacentric pairs (Figure 2c). A secondary constriction was observed on the second pair of metacentric chromosomes.

*M. simplex* showed the largest chromosome number of the genus, 2n = 36 (*n = 18*). It was composed by ten metacentric and eight submetacentric pairs (Figure 2d). The first metacentric and submetacentric chromosomes (pairs 1 and 11) were large and the remainders were from medium to small in size.

**Figure 3:**
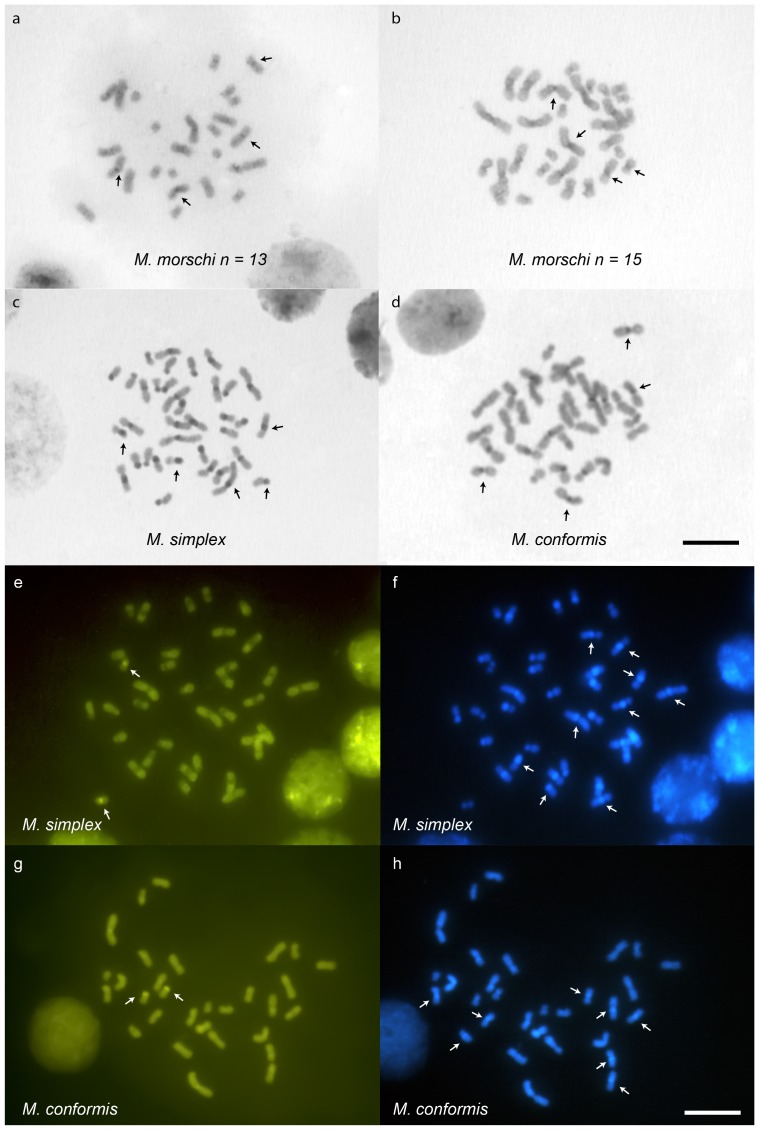
*Mycetophylax* metaphases submitted to C–banding technique and stained with fluorochromes. (a) C – banding in the worker metaphase of *M. morschi* 2n = 26, (b) *M. morschi* 2n = 30, (c) *M. simplex*, (d) *M. conformis* denoting the heterochromatic positive bands (dark grey as indicated by the arrows). Metaphase of (e, f) *M. simplex* and (g, h) *M. conformis* stained with fluorochromes CMA_3_ and DAPI, respectively. The white arrows indicate the positive staining for CMA_3_. DAPI positivity was in agreement with the C – banding pattern (some positive bands are indicated by white arrows). Bar  = 5 µm.

The results of chromosome banding and staining are shown in Table 1. The species showed different patterns of heterochromatin distribution (Figure 3). In *M. morschi* karyotypes, the heterochromatin is quite evident and can be distinguished in a few chromosomes in the centromeric region (Figure 3a and b, dark grey as indicated by the arrows). Moreover, *M. conformis* and *M. simplex* showed conspicuous heterochromatin blocks in the centromeric and pericentromeric regions (Figure 3c and d, dark grey as indicated by the arrows). The sequential fluorochrome staining revealed positive GC rich blocks (CMA_3_^+^) in only one pair, on the telomeric region, in *M. conformis* and on the pericentromeric region in *M. simplex* (Figure 3e, g – white arrows). DAPI showed general banding pattern coincident with the C–bands, indicating that the heterochromatin is AT rich (Fig. 3f and h – shiny blue as indicated by white arrows). *M. morschi* did not show any GC or AT rich regions in either karyotype, since the chromosomes were stained uniformly (data not shown).

**Table 1:** Cytogenetic data of the *Mycetophylax* species. Summary of chromosome.

	Chromosome number (n)	Chromosome morphology			C–banding positive blocks			Fluorochrome staining	
		M	SM	A	C	PC	SA	AT+ bands	GC+ bands
***M. simplex***	18	10	8	no	yes	yes	yes	yes	yes
***M. conformis***	15	11	4	no	yes	yes	yes	yes	yes
***M. morschi***	15	9	5	1	yes	no	no	no	no
***M. morschi***	13	9	3	1	yes	no	no	no	no

number, chromosome morphology and banding patterns of observed karyotypes of all the specimens analyzed here.

M: metacentric; SM: submetacentric; A: acrocentric.

C: centromeric; PC: pericentromeric; SA: Short arm.

### Chromosome evolution

**Figure 4:**
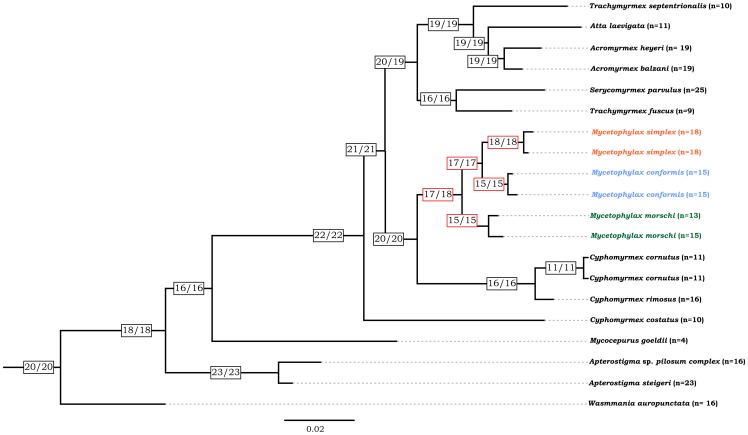
Chromosome number evolution and inferred ancestral chromosome state in the genus *Mycetophylax* (red boxes) inferred under Bayesian and Maximum likelihood optimization, including other Attini ants and an outgroup. Boxes at the nodes present the inferred ancestral haploid chromosome number for each node by Bayesian and ML analysis, respectively. Numbers at the tips are the known haploid chromosome numbers of species.

The results obtained in the analysis of chromosome evolution suggested that the best supported model of the process underpinning chromosome change was the hypothesis with constant gain, loss and duplication (Table 2). The rate parameters estimated in the best model were 16.52 for loss (*δ*), 7.01 for gain (*λ*) and 0.40 for duplication (*ρ*). The total inferred chromosome loss events were 181.41, gain 72.91 and duplication 1.70. These results revealed the occurrence of polyploidization events and suggested that whole karyotype duplication could have occurred during the chromosome evolution of these species. The main events inferred were loss (fusion) and gain (fission), which showed PP>0.5. In the Bayesian analysis, the haploid chromosome number at the most recent common ancestor (MRCA) of *Mycetophylax* with highest posterior probability (PP) was *n = 17* and in the ML analysis the most likely haploid number was *n = 18* (Figure 4, see Figure S1 for details).

**Table 2:** AIC scores and likelihood estimates for the data set analyzed for each model implemented by ChromEvol software.

Models	Log-likelihood	AIC scores
Gain, Loss and Duplication constant*	−56.91	119.8
Gain and Loss constant, no duplication	−58.01	120
Gain, Loss and duplication constant, Gain and Loss depend linearly on the current chromosome number	−55.67	121.3
Gain and Loss constant, Gain and Loss depend linearly on the current chromosome number, no duplication	−57.37	122.7

*Best fitting model.

To describe an evolutionary scenario for chromosome evolution inferred by ChromEvol to *Mycetophylax*, we focused on the haploid chromosome numbers estimated in the Bayesian method, since this method provides posterior probabilities (PP) as a statistical parameter. From the MRCA of the *Mycetophylax* species, the chromosome number decreased, becoming *n = 15* (PP = 0.43) and subsequently, n = 13 in the branch leading to *M. morschi*. Likewise, in the branch leading to *M. conformis*, the chromosome number decreased to *n = 15* (PP = 0.63). In the case of *M. simplex*, the haploid chromosome number increased to *n = 18* (PP = 0.74).

**Figure 5:**
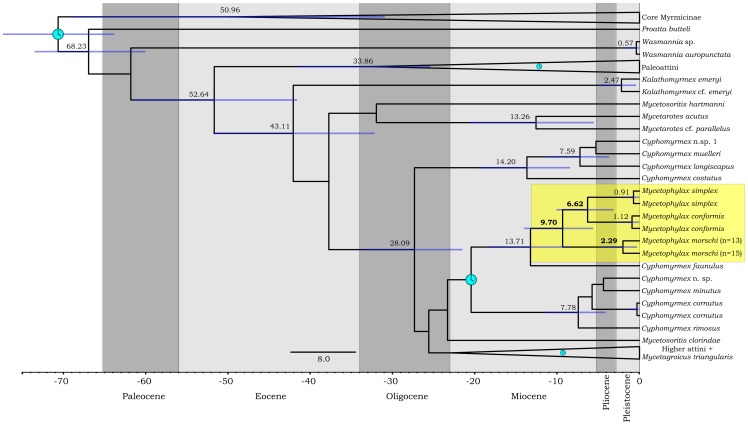
Bayesian time–calibrated maximum clade–credibility tree using a relaxed clock. Two calibration points are indicated with blue clocks and the third and fourth are suppressed within the Paleoattini clade and Higher attini clade (for calibration point details see Schultz and Brady, 2008). The numbers on the upper branches are the inferred age of the nodes (shown in red for *Mycetophylax*), while the 95% credibility intervals are indicated as blue bars on the nodes.

The Bayesian time–calibrated tree allowed us to infer that the *Mycetophylax* species diverged from *Cyphomyrmex* during the Miocene, around ∼13 Ma (95% CI = 8.49–18.91, Figure 5). This divergence was probably followed by chromosome changes. Considering the three species of *Mycetophylax*, *M. morschi* split early, around 9.1 Ma (95% CI = 5.75–14.34), whereas *M. conformis* and *M. simplex* split around ∼6.62 Ma (95% CI = 3.22–10.32). The initial emergence of *M. morschi* karyotypes was estimated to have occurred during the Pleistocene, around ∼2.29 Ma (95% CI = 0.31–4.72).

## Discussion

We detected wide chromosomal variability among the species of the genus *Mycetophylax*. The three species analyzed showed different karyotypes and, in fact, we verified two different diploid chromosome numbers for *M. morschi*. However, the karyotypes did not show significant geographic structuring, since they were found in both the northern and southern occurrence areas of this species. In three sampled localities, Angra dos Reis beach in the State of Rio de Janeiro and Mostardas and Chuí beaches in the State of Rio Grande do Sul (see Figure 1, Table S1), we detected the karyotypes *n = 13* and *n = 15*, though not living sympatrically on the same beach.

Despite the large number of colonies analyzed, we did not find hybrid karyotypes. Intermediate karyotypes (hybrid karyotypes or heterokaryotypes) would be expected if there was still gene flow among the *M. morschi* citotypes. Besides, due the differences with respect to chromossome number among karyotypes would be expected heterokaryotypes that show chromosomes that do not exactly match in pairs. Since this unmatched chromosome were not observed in the colonies analyzed we conclude that the two *M. morschi* citotypes are in fixed populations and should be treated as separate species (to be further taxonomically described). The role of chromosome changes in the speciation has been extensively reported in literature [1], [28], [29], and it has been proposed to accelerate evolution of the species [30]. One hypothetical scenario that could explain the observed differences in karyotype number suggests that changes in chromosome number evolve gradually overtime by multiple events of rearrangements that culminate in different races due to fixation of a single or few chromosomal changes, followed by extinction of intermediate karyotypes [31]. The observed citotypes of *M. morschi* may be a result of this step-by-step mechanism of chromosome evolution. Moreover, this is in agreement with the general rule that changes in the karyotype occurs throughout species diversification in ants. Usually when species from one genus are cytogenetically analyzed they show polymorphic karyotypes regarding both number and morphology [2].

The most commonly invoked mechanism for chromosome evolution in ants is “the minimum-interaction theory” proposed by Imai et al. [5]. According to this theory, karyotype changes tend toward increasing the number of chromosomes in order to minimize the threats of deleterious rearrangements due to the interaction of chromosomes within the nucleus. In general, this model predicts an increase in chromosome number due to centric fission, followed by chromatin addition (mainly heterochromatin) or pericentrometric inversions (for details see [5], [32]). Therefore, in the course of evolution the number of chromosomes will increase in number and reduce in size. Although the minimum interaction theory does not disregard fusions, this chromosome rearrangement is considerate rare and fixed or positively selected when it bring about short-term advantages [33].

Considering the minimum interaction theory, the ancestral karyotype of *M. morschi* would be *n = 13*, reaching *n = 15* by mean of fission rearrangements. However, based on our analysis of chromosome evolution, the recovered ancestral haploid chromosome number between karyotypes of *M. morschi* was *n = 15*, suggesting that the karyotype *n = 13* probably arose due to tandem fusion from the karyotype with *n = 15* chromosomes. The karyotypes do not show any absence of the medium size chromosomes, which would be expected in the case of centric fission from *13* to *15* haploid chromosomes, or acrocentric chromosomes in the karyotypes *n = 15*, which could have occurred in the case of centric fusion from *n = 15* chromosomes to *n = 13*. Likewise, the contemporary haploid chromosome number of *M. conformis* seems to be produced by fusion, decreasing from *n = 17* to *n = 15*. The estimated ancestral haploid chromosome number between *M. confomis* and *M. simplex* was *n = 17*, which is also the ancestral state estimated for the genus *Mycetophylax*. Thus, the karyotype number verified for *M. simplex* may have evolved due to centric fission instead of fusion, since it shows a haploid number of 18 chromosomes.

Several studies that evaluate karyotype evolution within an ant genera advocate in favor of centric fission as the main chromosomal rearrangement determining karyotypes, e.g. regarding Ponerinae ants, the suggestion is that chromosome changes occurred in the evolution of the genera *Odontomachus* and *Anochetus* [34]. The authors explained that centric fission is the principal evolutionary force acting on the karyotypes of *Odontomachus*, resulting in a larger, more stable karyotype mainly composed of subtelocentric chromosomes, compared with *Anochetus*, which is characterized by extreme karyotype diversification ranging from *n = 12* to *n = 23* and mainly composed of metacentric chromosomes. However, tandem fusion has been proposed to drive karyotype differentiation in a few cases (reviewed by [2]). In *Myrmecia pilosula*, this chromosome rearrangement was used to explain the origin of a long metacentric chromosome through the fusion of a subtelocentric and an acrocentric chromosome [35]. Chromosome fusion was also suggested to be involved in the genus *Acromyrmex*, due the decrease in the chromosome number, from n = 19 to n = 18, in *Acromyrmex ameliae* [36]. Notwithstanding, tandem fusion has also been reported to be involved in chromosome rearrangements for great number of other animals, including grasshoppers [37], [38]; wasps [39] and bats [40]. Our findings suggest that both fusion and fission may interplay during karyotype evolution in ants, promoting speciation by reducing or impairing gene flow. Chromosome changes are known to limit gene flow in parapatric and sympatric populations by means of gene isolations (unable to rearrange during meiosis) that can accumulate in consequence of rearrangements [31], [41], hence promoting population differentiation and speciation due to deficient recombination.

According to molecular phylogenetic analysis [17], *Mycetophylax* is divided into two major linages: one is composed only by the species *M. morschi* and the other comprises *M. conformis* and *M. simplex*. Since the last two species are more closely related to each other, the majority of metacentric and submetacentric chromosomes may be a characteristic shared by *M. conformis* and *M. simplex*. Furthermore, the pair of acrocentric chromosomes common to the karyotypes of *M. morschi* may be a symplesiomorphic chromosomal character retained from the ancestor that was lost in the lineage that diversified into *M. simplex* and *M. conformis*. On the other hand, this chromosome rearrangement could be one of the karyotypical characters that differentiate *M. morschi* from the others.

The C–banding technique and fluorochrome staining confirmed the cytotaxonomic groups distinguished by chromosome morphology analysis. Both karyotypes of *M. morschi* showed minimal amounts of heterochromatin and uniform fluorochrome staining. In contrast, *M. conformis* and *M. simplex* comprise a distinct group with intermediate to large amounts of AT-rich heterochromatin and a pair of chromosomes bearing a CG–rich region. The banding patterns shared by *M. conformis* and *M. simplex* suggest that their chromosomes underwent rearrangements following the split from a common ancestor related to *M. morschi*. Moreover, the amount of heterochromatin found in *M. simplex* is in agreement with the higher DNA content of this species. The 1C DNA amount estimated to *M. simplex* was 381.42 Mbp, whereas both karyotype of *M. morschi* and *M. conformis* have identical genome sizes with 312.96 Mbp [14].

The interspecific karyotype variability found among the species of the genus *Mycetophylax* could be associated with the biological environment where these species are restricted. As mentioned above, these species are confined to sand dune habitats along the Atlantic coast [12], [15]. This area is known to have been strongly influence during the Quaternary, having been remodeled due to periods of transgression and regression of the sea level [42]. Our results suggested that the genus *Mycetophylax* diverged from *Cyphomyrmex* during the middle Miocene (∼13 Ma) and diversified into the current species between the end of the Miocene and the beginning of the Pliocene. These periods are marked by deep modifications in the landscape that could facilitate the isolation of populations, culminating in the accumulation of chromosome mutations that could favor speciation and the evolution of new taxa. We suggest that an ancestor of these species was distributed along the Atlantic coast and later, due to transgressive movements of the sea, the geographic distribution was split by rising sea levels, producing barriers and sandy islands where the speciation process took place. The distinct karyotypes of *M. morschi* arose during the Pleistocene, a period extensively reported to have influenced the diversification of species in the Brazilian Atlantic Forest [43], which includes its coastline. Highly intra and inter–specific karyotype variability is also reported for the genus *Ctenomys* [44], a subterranean rodent that has habitat requirements and a distribution pattern similar to *Mycetophylax*. Thus, the sand dune environments on the Atlantic coast of Brazil and their geological history could have acted as a trigger for chromosomal rearrangements and the subsequent speciation in these areas.

This is the first comprehensive cytogenetic description and evolutionary analysis of an Attini genus based on molecular data and provides a baseline for future comparative and integrative studies. Based on our chromosome evolution approach and cytogenetic banding techniques, we hypothesized that fusions instead of fission could be involved in the chromosome evolution of the *Mycetophylax*. These chromosome rearrangements likely took place by involving complete genetic isolation of the two major lineages within *Mycetophylax* that therefore established their own evolutionary strategies. One of these lineages diversified into the *M. morschi* group complex and the other diversified into *M. simplex* and *M. conformis*. Overall, the results presented in this study confirm that tandem fusion could very well participate in chromosome ant evolution. Our karyological analysis based on phylogenetic framework suggests that some chromosome rearrangements may be more recurrent than previously thought. Besides, this integrative approach can be helpful to avoid misinterpretations on chromosome changes during species diversification. It is important that studies involving cytogenetic data within genus and between related genera are continued and that these studies take into account molecular phylogenic methods in the evaluation of the cytogenetic data of ants, as well in other taxa.

## Supporting Information

Figure S1 **Chromosome number evolution and inferred ancestral chromosome state in the genus** ***Mycetophylax*** **inferred under Bayesian and Maximum likelihood optimization with inferred frequency of fusion and fission events estimated throughout the phylogenetic tree.** Green numbers at the branches and tips represent the inferred frequency of gain events (fission) and purple loss events (fusion) that had a probability >0.5. The analysis was carried out including other Attini ants and *Wasmannia auropunctata* as outgroup (Myrmicinae subfamily). Boxes at the nodes present the inferred ancestral haploid chromosome number for each node by Bayesian and ML analysis, respectively. Numbers at the tips are the known haploid chromosome numbers of species.
(TIF)

Table S1 **Sampled localities and number of the colonies analyzed cytogenetically per collected site and species.** RS – Rio Grande do Sul state, SC – Santa Catarina state, PR – Paraná state, SP – São Paulo state, RJ – Rio de Janeiro state.
(XLS)

Table S2 **GenBank accession numbers of specimens used for phylogenetic inference in molecular clock analysis and to reconstruct the Bayesian tree used in the haploid ancestral state reconstruction and chromosome evolution analysis (in bold).**
(XLS)
