# Correction: The Role of Fusion in Ant Chromosome Evolution: Insights from Cytogenetic Analysis Using a Molecular Phylogenetic Approach in the Genus *Mycetophylax*

**DOI:** 10.1371/journal.pone.0095408

**Published:** 2014-04-10

**Authors:** The PLOS ONE Staff

There is an error in Table 1. During the production process, part of the table caption got merged with the information under the table. Please see the corrected Table 1 here.

**Table 1:** Cytogenetic data of the *Mycetophylax* species. Summary of chromosome number, chromosome morphology and banding patterns of observed karyotypes of all the specimens analyzed here.

	Chromosome number (n)	Chromosome morphology			C–banding positive blocks			Fluorochrome staining	
		M	SM	A	C	PC	SA	AT+ bands	GC+ bands
*M. simplex*	18	10	8	no	yes	yes	yes	yes	yes
*M. conformis*	15	11	4	no	yes	yes	yes	yes	yes
*M. morschi*	15	9	5	1	yes	no	no	no	no
*M. morschi*	13	9	3	1	yes	no	no	no	no

M: metacentric; SM: submetacentric; A: acrocentric.

C: centromeric; PC: pericentromeric; SA: Short arm.
