# Supplementary figures and images for: The Role of Fusion in Ant Chromosome Evolution: Insights from Cytogenetic Analysis Using a Molecular Phylogenetic Approach in the Genus Mycetophylax
Source: PLoS One. 2014 Jan 28;9(1):e87473. doi: 10.1371/journal.pone.0087473 (PMC3904993; doi:10.1371/journal.pone.0087473)

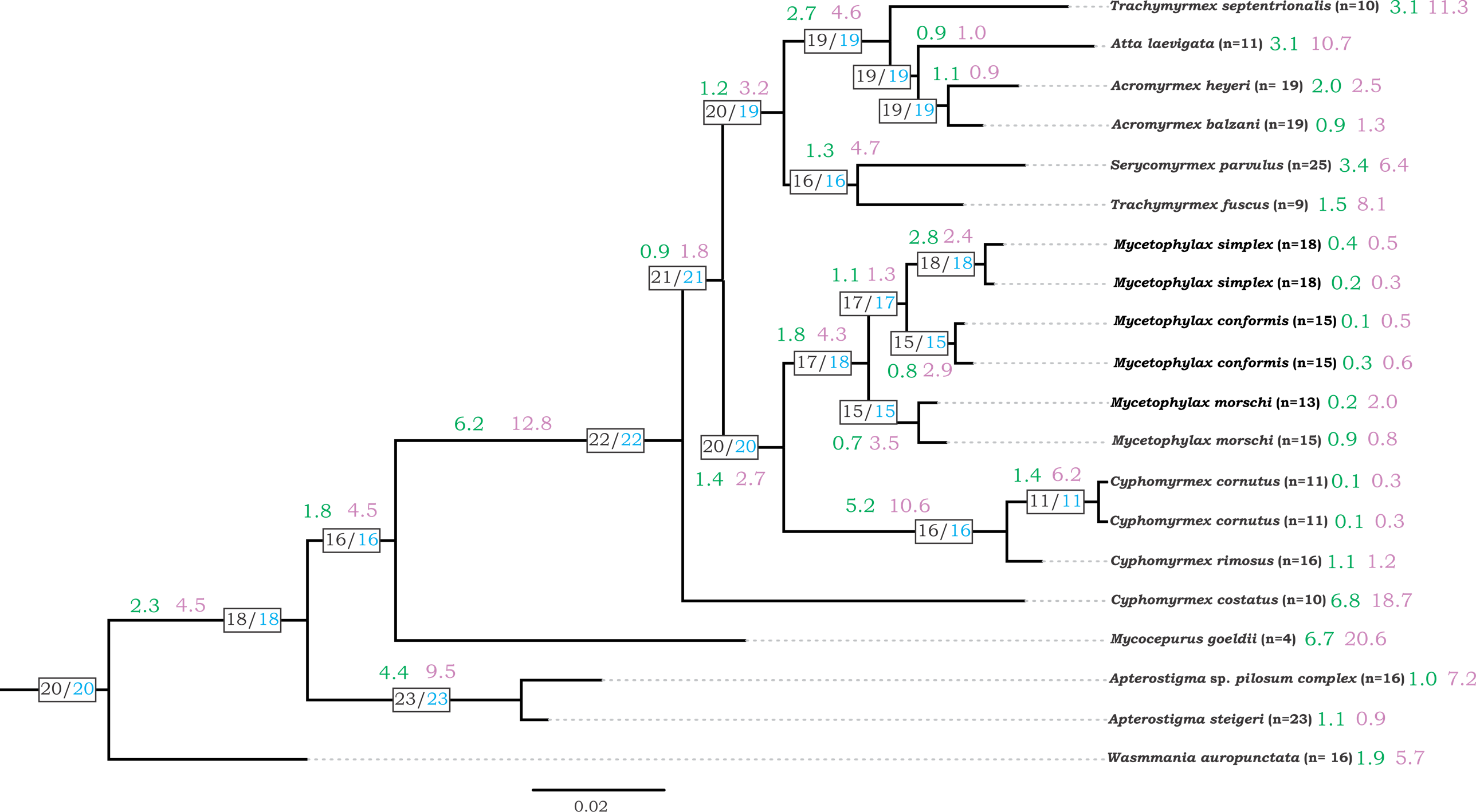

Supplement: Figure S1 — Chromosome number evolution and inferred ancestral chromosome state in the genus Mycetophylax inferred under Bayesian and Maximum likelihood optimization with inferred frequency of fusion and fission events estimated throughout the phylogenetic tree. Green numbers at the branches and tips represent the inferred frequency of gain events (fission) and purple loss events (fusion) that had a probability >0.5. The analysis was carried out including other Attini ants and Wasmannia auropunctata as outgroup (Myrmicinae subfamily). Boxes at the nodes present the inferred ancestral haploid chromosome number for each node by Bayesian and ML analysis, respectively. Numbers at the tips are the known haploid chromosome numbers of species. (TIF) [file pone.0087473.s001.tif]
